# The Illusion of Uniformity Does Not Depend on the Primary Visual Cortex: Evidence From Sensory Adaptation

**DOI:** 10.1177/2041669518800728

**Published:** 2018-09-27

**Authors:** Marta Suárez-Pinilla, Anil K. Seth, Warrick Roseboom

**Affiliations:** Sackler Centre for Consciousness Science, University of Sussex, Brighton, UK; Department of Informatics, University of Sussex, Brighton, UK

**Keywords:** perceptual uniformity, uniformity illusion, peripheral vision, tilt aftereffect

## Abstract

Visual experience appears richly detailed despite the poor resolution of the majority of the visual field, thanks to foveal-peripheral integration. The recently described uniformity illusion (UI), wherein peripheral elements of a pattern take on the appearance of foveal elements, may shed light on this integration. We examined the basis of UI by generating adaptation to a pattern of Gabors suitable for producing UI on orientation. After removing the pattern, participants reported the tilt of a single peripheral Gabor. The tilt aftereffect followed the physical adapting orientation rather than the global orientation perceived under UI, even when the illusion had been reported for a long time. Conversely, a control experiment replacing illusory uniformity with a physically uniform Gabor pattern for the same durations did produce an aftereffect to the global orientation. Results indicate that UI is not associated with changes in sensory encoding at V1 but likely depends on higher level processes.

## Introduction

Visual experience appears richly detailed despite the poor sensory precision of the majority (periphery) of the visual field. This topic has received considerable recent attention (Cohen, Dennett, & Kanwisher, 2016; Haun, Tononi, Koch, & Tsuchiya, 2017), with debate about the degree to which visual experience is in fact rich and the potential perceptual processes that may contribute to apparent richness. One recent study demonstrated a compelling example of how the rich detail within the high-precision central visual field alters peripheral perception—the uniformity illusion (UI; Otten, Pinto, Paffen, Seth, & Kanai, 2016). UI describes a phenomenon wherein apparent perceptual uniformity occurs when variable sensory stimulation is presented in peripheral vision, while the central visual field is presented with uniform stimuli. UI occurs for a wide variety of perceptual dimensions, including relatively low-level sensory features such as orientation or colour and higher level features such as density (see www.uniformillusion.com for examples).

We sought to examine the mechanisms underlying UI using perceptual adaptation. It is well established that exposure to a specific stimulus magnitude (like an oriented grating) causes perceptual aftereffects (e.g., tilt aftereffect [TAE]; Gibson & Radner, 1937). For visual orientation, perceptual aftereffects have been associated with specific changes in neural coding at the primary visual cortex (V1) and are localized in a retinotopic reference frame (Knapen, Rolfs, Wexler, & Cavanagh, 2010). Here, we utilize the spatial specificity of TAE to examine whether the apparent perceptual uniformity in UI can be attributed to changes in V1-based neural coding for visual orientation. Specifically, we presented participants with Gabor grids wherein the orientation of central elements was uniform, but the orientation of peripheral elements was variable—producing UI. At fixed test locations in the periphery of the grid, we presented a physical orientation that differed from the global illusory percept, thus putting local and global orientation in opposition. Following prolonged exposure to global illusory uniformity (UI), we contrasted whether the resultant TAE was consistent with the local, physical orientation or the illusory global orientation.

## Methods

### Procedure

**Figure 1.**
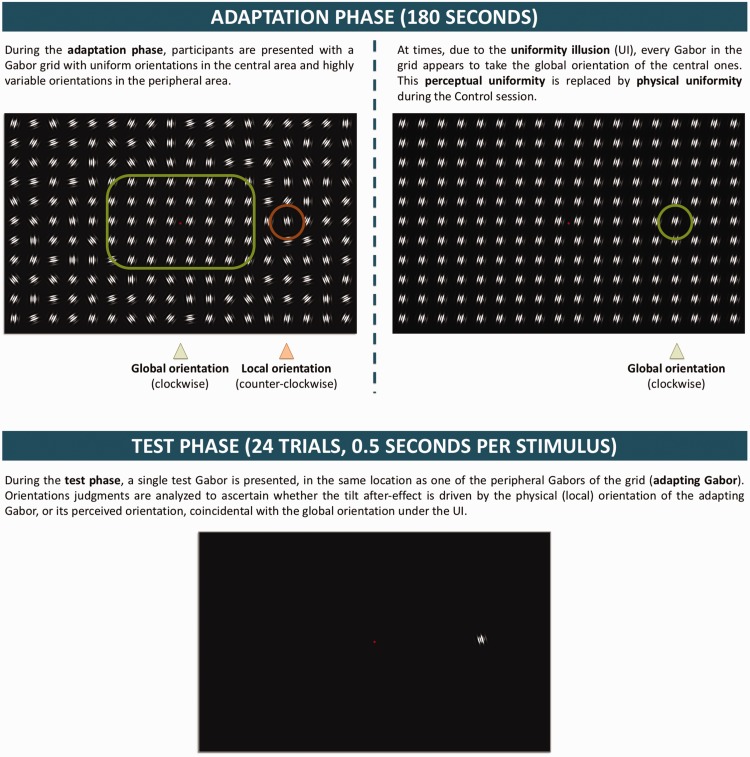
Experimental structure. During the adaptation phase, participants were presented with a Gabor grid wherein the central Gabors had a uniform orientation, while peripheral orientations were heterogeneous. Under UI, perceptual experience was that of a uniform pattern with all Gabors tilted like the central ones. This illusory percept alternated with a nonillusory, nonuniform percept at different times during adaptation. For a specific peripheral Gabor (adapting Gabor), physical and illusory orientation were always in opposition. The control session replicated the phenomenology of the illusion session, replacing perceived with physical uniformity at times in which the participant reported UI in the illusion session. The test phase had 24 trials, wherein participants reported the tilt of a single peripheral Gabor whose location coincided with the adapting Gabor.

The experiment had two parts: illusion session and control session. Each session contained six blocks, and each block had an adaptation phase and a test phase (Figure 1). A practice block was run before the illusion session to familiarize participants with UI.

#### Illusion session

Each block began with an adaptation phase, in which participants were presented with a grid of Gabor patches suitable for producing the UI, affecting the apparent orientation of peripheral elements: All Gabors in the central area had a uniform orientation, whereas orientation of the peripheral Gabors was heterogeneous. Gaze-contingent stimulus presentation ensured that each Gabor was presented to a specific retinal location, as the entire pattern was removed if the participant’s gaze deviated from central fixation by more than 1.5 degrees of visual angle (dva)—a tolerance threshold equivalent to half the size of each cell of the grid. Adaptation lasted 180 seconds, but because the stimulus was removed when fixation lapsed, actual exposure time could be shorter.

Participants reported the experience of illusory uniformity by pressing a key when all Gabors appeared to take a uniform orientation.

The test phase had 24 trials, separated by a pseudorandom interval of 1,000 to 1,500 ms. In each trial, a single Gabor (test Gabor) was presented for 500 ms at a specific peripheral location, coinciding with the position of a specific Gabor during adaptation (adapting Gabor). Participants reported if the test Gabor was tilted clockwise (CW) or counterclockwise (XCW) from vertical.

#### Control session

The control session also had six blocks, each built to replicate the phenomenology of a homologous block of the illusion session but replacing illusory for physical uniformity during the adaptation phase.

During the adaptation phase in the illusion session, an empty background was presented whenever the gaze-contingent mechanism removed the adapting pattern. The same pattern of stimulus presentation and removal was replicated in the control session. The stimulus was additionally removed whenever fixation lapsed in the control session. At any other time, the presentation displayed one of two patterns, differing only in the orientation of peripheral Gabors. The first was identical to the pattern presented in the illusion session and was displayed at times in which the participant had *not* reported UI during adaptation in the illusion session. At times during which the participant had reported UI, the presented pattern was one in which all Gabors had the same *physical* orientation, consistent with the desired illusory orientation during the illusion session. Thus, physical uniformity was inserted at the times in which illusory uniformity had been reported in the illusion session. Participants were not informed that this would occur.

The test phase was identical to that in the illusion session: The location and orientation of the test Gabor in each trial was identical, as well as its test latency (time between the end of the adaptation phase and stimulus onset).

### Stimuli

Stimuli were displayed on dark grey background (1.96 cd/m^2^). A red fixation dot (8.34 cd/m^2^, 0.42 dva diameter) showed constantly on the screen centre.

#### Gabor patches

Each Gabor consisted of a sine-wave luminance grating with Michelson contrast of 1, 0° phase, and spatial frequency of 1.66 cycles per dva, and a two dimensional Gaussian envelope with a sigma of 0.43 dva.

#### Adapting pattern

The adapting pattern spanned the entire screen and consisted of a 13 × 17 grid formed by invisible square cells measuring 3 dva per side (Figure 1). Each Gabor was presented in the centre of each cell. The central area spanned 15 dva horizontally and vertically, encompassing all cells belonging to rows 5 to 9 and columns 7 to 11. All central Gabors had the same orientation, which could be one of two values, each for half the blocks of one session: −15° (global clockwise tilt [GCW]) or 15° (global counterclockwise tilt [GXCW]). The orientations of peripheral Gabors were sampled from a discrete uniform distribution centred on the global orientation and ranging 70° (35° to each side). Thus, mean orientation was the same for central and peripheral Gabors and matched the global orientation perceived under UI.

Two peripheral Gabors of the pattern (adapting Gabors) corresponded to the positions in which the test Gabors would be displayed during the test phase: They were located along the middle (7th) row, at 12.02 dva left and right of the screen centre (columns 5 and 13). Both had the same nonrandomized local orientation, which was the opposite of the global orientation of the block: either 15° (local counterclockwise tilt [LXCW]) or −15° (local clockwise [LCW]).

Henceforth, we give the label adapting condition CX to the presentation pattern wherein the local orientation of the adapting Gabor is clockwise, and the global orientation of the pattern is counterclockwise (LCW, GXCW). Conversely, we will refer to the pattern with LXCW and GCW orientations as adapting condition XC. Both conditions occurred equally frequently during the experiment.

As described above, during the control session, the adapting pattern was replaced by a physically uniform pattern at those times during which participants had reported UI in the illusion session. In these instances, *every* Gabor in the pattern (including the adapting Gabors) took the global orientation.

#### Test Gabors

A single test Gabor was presented per trial, matching the position of one of the two adapting Gabors. Test Gabors were displayed in the left and right hemifield with equal frequency per block and could take one of eight equally frequent orientations: −12°, −5°, −2°, −1°, 1°, 2°, 5°, and 12° (negative values indicate CW tilt). Thus, test orientations were always intermediate between global and local orientations (−15°, 15°).

### Participants

Participants were recruited through online advertisement, were older than 18 years, and reported normal or corrected-to-normal vision. This study received ethical approval by the Research Ethics Committee of the University of Sussex.

### Apparatus

Experiments were programmed in MATLAB 2016a (MathWorks Inc., Natick, MA, USA) and displayed on a LaCie Electron 22BLUE II 22″ with screen resolution of 1,024 × 768 pixels and refresh rate of 100 Hz. Eye-tracking was performed with EyeLink 1000 Plus (SR Research, Mississauga, Ontario, Canada) at sampling rate of 1000 Hz, with level desktop camera mount. Head position was stabilized 43 cm from the screen using chin and forehead rest. Calibration of the eye-tracker was performed at the beginning of each block with a standard five-point grid and a maximal average error of 0.5 dva.

### Statistical Analysis

Psychometric curve fitting was performed in MATLAB 2017b, using Palamedes toolbox, version 1.8.1 (Prins & Kingdom, 2009). A cumulative Gaussian curve was fitted by the method of maximum likelihood to the proportion of “XCW” responses per test Gabor orientation, separately for each participant and session/condition (depending on the specific analysis). The threshold (α) for 0.5 proportion of XCW responses and the slope of the curve (β) were free parameters (starting values: α = 0°, β = .04), while guessing (γ) and lapse rate (λ) were fixed at zero.

Bayesian statistics were conducted on JASP (JASP Team, 2017, version 0.8.3.1). For Bayesian *t* tests, we employed as prior distribution Cauchy(0, $\frac{1}{2}\sqrt{2}$) for two-sided predictions, or a folded Cauchy(0, $\frac{1}{2}\sqrt{2}$) for one-sided predictions (Measure 1 < Measure 2). Likewise, for Bayesian Pearson correlations, we employed a uniform distribution U(−1,1) for two-sided or U(0,1) for one-sided (positive) predictions. For each contrast result, the prior utilized is indicated by the formulated prediction and the subscripts in BF_10_ (two-sided) or BF_−0_ /BF_+0_ (one-sided).

## Results

Thirty participants volunteered for the experiment: 23 female; mean age was 21.6 years. To ensure sufficient exposure to the adapting pattern, we excluded blocks wherein the pattern had been displayed for less than two thirds of the adaptation phase (<120 seconds), due to gaze-contingent stimulus removal. In such cases, the corresponding blocks from both control and illusion sessions were removed to maintain balance. This caused exclusion of 32.78% blocks (118/360), including the entire data sets from five participants. Furthermore, because our analyses compared responses across adapting conditions (CX/XC), two additional participants were excluded as all their valid blocks were of only one condition. Results presented here correspond to the remaining 23 participants. Overall, results for all 25 participants with valid blocks were very similar to this counterbalanced sample (see Supplementary Materials, Section S5).

### Adaptation Phase

Average exposure time to the adapting pattern per block was 164.13 and 149.47 seconds for the illusion and control sessions: 91.18% and 83.04% of the adaptation phase, respectively. The lower proportion in the control session was expected as pattern removal occurred whenever it had in the illusion session, in addition to times of improper fixation in the control block.

Perceived uniformity was reported, on average, for 43.48 seconds in the illusion session, 26.77% of the time of pattern presentation (minimum 0.55%, maximum 72.23%). The proportion of time of perceived uniformity during the control session was similar to that for the illusion session: 28.41% (minimum 0.59%, maximum 78.42%, Bayesian paired-samples *t* test: BF_01_ = 2.733—anecdotal evidence for the null hypothesis). Physical uniformity in the control session was reported as perceptually uniform 68.13% of the time; by contrast, the nonuniform pattern was reported as uniform only 9.24% of the time. Possibly, presentation of a truly uniform pattern at times shifted a subjective criterion for uniformity by comparison, leading to more conservative reports in the control sessions.

### Hypotheses and Measurements

The experiment placed adaptation to illusory and physical orientation in opposition to disambiguate between two competing hypotheses:

1. The perceived orientation under UI has no effect on tilt adaptation; the TAE is driven solely by the physical orientation of the adapting Gabor.
2. The global orientation perceived for the entire pattern (including the adapting Gabor) under UI can produce a TAE.

To decide between hypotheses, data were analysed to ascertain the direction of the adaptation-induced bias. We calculated the proportion of XCW reports per test Gabor orientation and obtained the best fitting cumulative Gaussian psychometric curve. The point of subjective equality (PSE) was defined as the test orientation at which 50% reports are XCW. Because CW orientations have (conventionally) negative sign and vice versa, negative PSE indicates a XCW bias, and positive PSE indicates a CW bias.

During the illusion session, a TAE driven by (i.e., away from) the local orientation of the adapting Gabor would imply physical adaptation, while a global-driven TAE would indicate adaptation to illusory orientation. During the control session, both local- and global-driven TAE are compatible with physical adaptation, as the adapting Gabor physically takes the global orientation at times of reported illusory uniformity in the illusion session.

By calculating participants’ PSE per adapting condition, we obtained two measurements:

1. PSE_CX_ and PSE_XC_. For a local-driven TAE, responses for adapting condition CX should exhibit a XCW bias compared with condition XC (PSE_CX_ < PSE_XC_), and the reverse should happen for a global-driven TAE.
2. dPSE = PSE_CX_–PSE_XC_. We employ this as a summary measure indicating the overall direction of the bias. A negative dPSE indicates a predominance of local-driven TAE (PSE_CX_ < PSE_XC_) consistent with physical adaptation to the local orientation, while a positive dPSE indicates a global-driven TAE, consistent with adaptation to the illusion (or to the physical replication of the illusion during the control session).

### TAE Is Driven by Physical, Not Illusory Orientation

#### Overall effect

##### Illusion session

**Figure 2.**
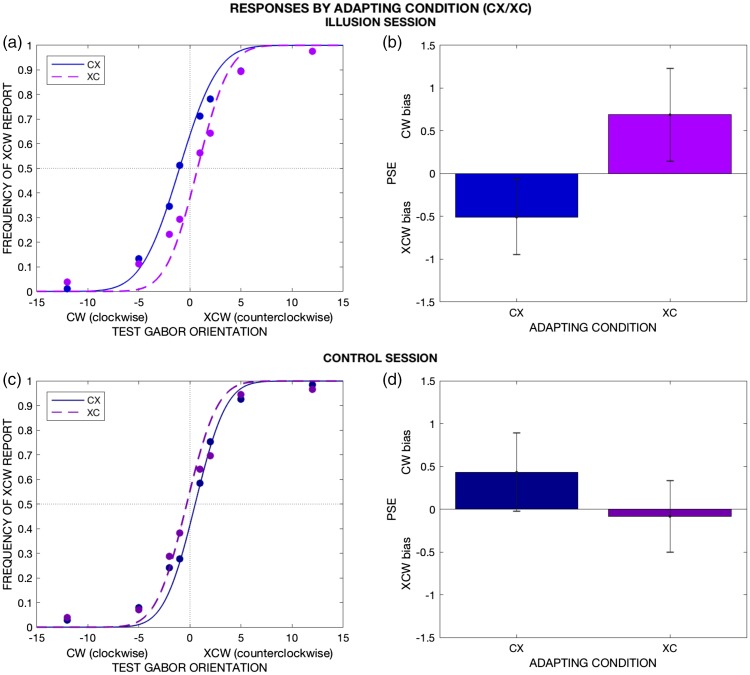
Response patterns by adapting condition. (a) and (c) presents the sample’s proportion of counterclockwise (XCW) reports per test Gabor orientation, separated by adapting condition, during the illusion (a) and control (c) sessions. The dotted lines show the best cumulative Gaussian fit for the psychometric curve of each condition, fitted on the sample’s pooled data (*N* = 23). (a) and (c) are included for illustrative purposes only, as the PSEs obtained for analysis were computed separately for each participant’s data: These results are depicted in (b) and (d), where the bar heights represent the average point of subjective equality (PSE), computed separately per participant, session, and condition. The error bars represent the between-participant standard error. (b) Illusion session. PSEs for both adapting conditions reflect a bias *away* from local orientation (local-driven TAE). (d) Control session. On average (d), responses show a global-driven TAE in CX and are unbiased in XC. These results show that perceived (illusion) and physical (control) uniformity behave differently, suggesting that the TAE is always driven by the physical orientation, even when that orientation is unseen under UI.

Figure 2(a) presents the average proportion of XCW reports per test Gabor orientation during the illusion session, separated by adapting condition (CX or XC). For illustration purposes, it shows cumulative Gaussian curves fitted to the pooled data. However, for analysis, we fitted each participant’s responses separately: Individual fits are detailed in the Supplementary Materials, Section S2. Individual PSEs for each adapting condition are summarized in Figure 2(b). On average, PSE_CX_ = −0.502° and PSE_XC_ = 0.687° reflected a XCW and CW bias, respectively: dPSE = −1.197° (PSE_CX_ < PSE_XC_ Bayesian paired-samples *t* test: BF_−0_ = 3.057) indicated a local, physical-driven adaptation.

##### Control session

In the control session, the adapting Gabor physically takes the global orientation of the pattern during times of reported uniformity in the illusion session. If adaptation is produced by physical orientation, we should observe a more global-driven TAE compared with the illusion session: dPSE_IL_ < dPSE_CO_. Conversely, if perceived orientation under UI causes adaptation, we should not see a difference between perceived and physical uniformity: dPSE_IL_ = dPSE_CO_.

Results indicate predominance of global-driven TAE during the control session (Figure 2(d)): PSE_CX-CO_ = 0.433°, PSE_XC-CO_ = −0.083°, dPSE_CO_ = 0.516°. A Bayesian paired-samples *t* test comparing dPSE in both sessions was consistent with physical-driven adaptation: dPSE_IL_ < dPSE_CO_, BF_−0_ = 7.476. Therefore, the absence of a global-driven TAE in the illusion session was not simply because the global pattern of orientation was insufficient to induce TAE; rather, the illusory (but not the physical) global pattern was insufficient to induce TAE.

The overall predominance of global-driven TAE in the control session, despite presentation of the uniform pattern for only ∼27% of time, may be related to a putatively stronger adaptation during this time due to the adjacent Gabors, which then take the global orientation, contributing to the receptive field(s) where the test Gabor will be later presented. Note, however, that the size of each grid cell (3 dva) is larger than the diameter of most receptive fields at V1 (around 1 dva; Bentley & Salinas, 2009), and the relationship between stimulus size and TAE strength is not always intuitive (Harris & Calvert, 1985; Parker, 1972). Another possibility involves extraclassical receptive field effects exerted by the global surround on the adapting Gabor when the latter takes the global orientation (iso-orientation surround suppression; Chen, Chen, Gao, Yang, & Yan, 2015). Whatever the contribution of these effects, they act differently on physical compared with illusory iso-orientation, in the manner expected for low-level processing of the former, but not the latter.

In the Supplementary Materials (Sections S2 and S4), we reanalyse the data set based on raw responses, rather than PSE from fitted curves. Both approaches show the same pattern of results, indicating that choices related to curve fitting and goodness-of-fit of psychometric curves do not significantly affect our analyses.

#### Time-dependent effect

Overall, responses in the illusion and control session fit the hypothesis that TAE under UI is only driven by physical and *not* illusory orientation. However, in the illusion session, UI is perceived during only ∼27% of pattern exposure, on average. Thus, it could be argued that a global, illusion-driven TAE might have been present, but undetected in the overall results, overshadowed by the local-driven TAE at times when UI is not perceived. This possibility seems unlikely because responses in the control session (with uniformity also presented ∼27% of time) *do* show an influence of the global-driven TAE. Thus, such a possibility could only hold if the TAE driven by illusory orientation was weaker than that caused by physical orientation. To rule out this possibility, we examined the data from the illusion sessions for evidence of exposure time-dependency of the TAE magnitude. Because the TAE is time-dependent (Patterson, Wissig, & Kohn, 2013), if illusion-driven adaptation was in fact present, we should find evidence for a shift towards more global/less local TAE with longer times of perceived uniformity.

##### Illusion session

If the TAE is driven *only* by physical orientation, in the illusion session, we should expect independence from time of perceived uniformity. Conversely, if the perceived orientation under UI causes adaptation, the response pattern should shift from predominantly local-driven towards more global-driven for longer time of perceived uniformity. We can assess this potential shift by examining dPSE. As stated above, negative dPSE indicates predominance of local-driven TAE and positive dPSE global-driven TAE. Thus, in the presence of illusion-driven adaptation, dPSE should correlate positively with time of perceived uniformity.

**Figure 3.**
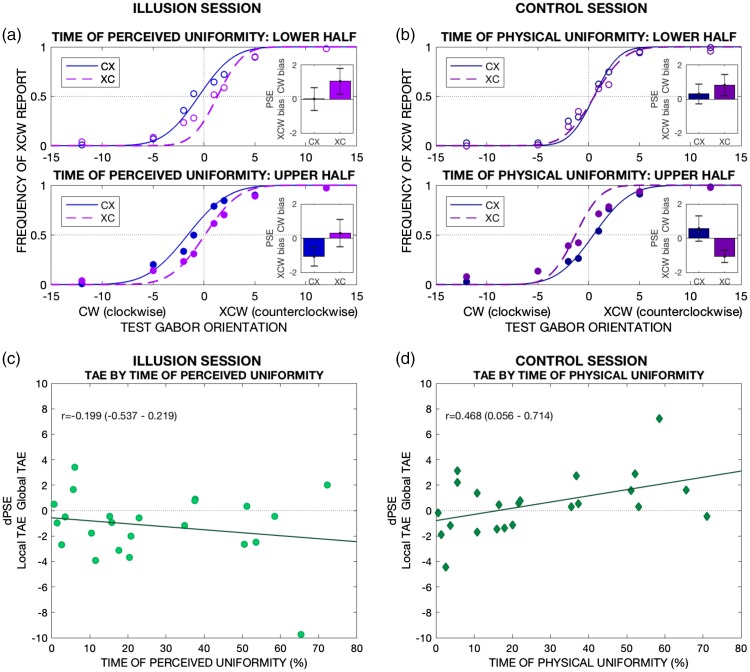
TAE by time of uniformity. Physical, but *not* perceived uniformity, causes a shift towards global-driven TAE in a time-dependent manner. (a and b) Participants are classified into two groups according to whether their average time of uniformity is below (lower half) or above (upper half) the sample’s median and depict each group’s average responses by adapting condition in the illusion (a) and control (b) session. For illustration purposes, a psychometric function fitted to the pooled data is shown in the main figures; however, all analyses are based on psychometric functions fitted to each participant’s data—the group average PSEs of these functions are shown in the insets. In the illusion session, PSEs indicate local-driven TAE regardless of time of perceived uniformity (except for condition CX in the lower half group, which shows no noticeable TAE overall). In the control session, the TAE shifts to global-driven for longer presented physical uniformity. (c and d) The correlation between each participant’s average time (%) of uniformity and dPSE during the illusion (c) and control (d) session is presented. For (c), the relationship is established with time of perceived uniformity, and for (d), the relationship is established with time of physical uniformity. Only for the latter (d) do we observe the predicted positive correlation with time, indicative of a shift towards more global TAE. PSE = point of subjective equality; TAE = tilt aftereffect.

As time measure, we employed the proportion of perceived uniformity (over time of pattern presentation) for conveying the balance between local and (putative) global effects. We analysed the bivariate correlation between time of perceived uniformity and dPSE (Figure 3(c)). Pearson’s correlation coefficient and 95% credible intervals were *r* = −.199 (−.537, .219), with moderate evidence against a positive correlation: BF_+0_ = 0.146.

Therefore, evidence opposed a positive association between time of perceived uniformity and a trend towards more global-driven TAE, thus opposing predictions expected for illusion-based adaptation.

##### Control session

For the control session, we performed analogous analyses as for the illusion session, but with time of physical instead of perceived uniformity.

Because global uniformity is a physical stimulus in this session, a time-dependent shift from local to global-driven TAE should be expected regardless of the capacity of illusory orientation to induce a TAE. Thus, this control session acts as a sanity check to rule out that the failure to find time-dependency in the illusion session was simply due to insufficient exposure to the global pattern—even in the cases of longest time of uniformity.

We performed a Bayesian bivariate correlation between individual average time of physical uniformity and dPSE (Figure 3(d)). Pearson’s correlation coefficient was *r* = .468 (95% credible intervals .056, .714), showing moderate evidence for a positive correlation: BF_+0_ = 5.546.

Thus, physical uniformity presented for durations equivalent to the reported illusory uniformity was sufficient to observe a shift towards a global-driven TAE.

## Discussion

The UI is a striking phenomenon in which experience across the whole visual field is modified by higher precision foveal information, yet its underlying mechanisms remain unknown. Using a version of UI with oriented Gabor patches, we found that UI does not produce an orientation adaptation aftereffect consistent with the illusory percept. Instead, orientation aftereffects only ever followed the (local) physically presented orientation. This suggests that the UI, at least in orientation, arises from higher level (higher than primary visual cortex) perceptual processes.

It has been suggested that the UI may result from predictive processing operations in the visual hierarchy (Otten et al., 2016). In a hierarchical predictive coding scheme, perception arises from the interaction of bottom-up sensory signals with top-down expectations generated in higher cortical areas (Friston, 2005; Rao & Ballard, 1999). Prediction error is determined by the discrepancy between bottom-up sensory signals and the top-down predictions and propagates through the sensory hierarchy to update the internal world model. Although the interplay between neural signatures of sensory adaptation and predictive coding is not fully understood (Symonds et al., 2017), evidence indicates that top-down expectations produce activity changes in the visual cortex also specifically for orientation-selective neurons in V1 (Schummers, Sharma, & Sur, 2005), with adaptation adjusting the relative weight of bottom-up and top-down signals in relation to their precision (Malmierca, Anderson, & Antunes, 2015). Under this framework, UI may be conceptualized as the result of high-precision foveal signals being given more weight in forming perceptual predictions for the presented pattern, possibly in combination with a prior for perceptual uniformity for the entirety of the visual field. After a period of exposure, adaptation renders low-precision peripheral signals weaker still, until eventually they become unable to overcome the central-based prediction (Otten et al., 2016).

Our results suggest that if UI does result from such predictive operations, the locus of influence of the feedback does not reach primary visual cortex, as illusory uniformity produced no measurable adaptation effect.

What, then, is the neural basis of UI? UI might be an instance of perceptual filling-in, a phenomenon whereby a visual attribute like colour, luminance, or texture is perceived in a region of the visual field even though it only exists in the surround (Komatsu, 2006). However, unlike typical instances of uniform spread of colour or luminance, in our orientation UI, the distinction between background and grid elements persists, and the illusion selectively informs the appearance of the individual Gabors. The process may be similar to texture filling-in or involve texture processing in a broader sense. Notably, several neurophysiological and neuroimaging experiments have reported changes in neural activation in early visual areas that correlate with perceptual filling-in; however, while for colour or luminance, this correlate has been seen at V1 (Hsieh & Tse, 2010), for texture filling-in, it has only been observed at V2 and above (De Weerd, Gattass, Desimone, & Ungerleider, 1995; Komatsu, 2006), in agreement with our results.

UI also exhibits similarities with crowding, as a context-dependent alteration of peripheral perception. Like UI, crowding arises for different low- and high-level dimensions and at several stages of the visual system, involving V2 and above (Whitney & Levi, 2011), for instance, tilt adaptation to the veridical orientation is present for crowded, indistinguishable stimuli (He, Cavanagh, & Intriligator, 1996). Crowding has been likened to texture perception (Parkes, Lund, Angelucci, Solomon, & Morgan, 2001). However, as a fundamental difference with crowding, in UI, peripheral phenomenology is not a mixture of adjacent stimuli, but the replacement of peripheral appearance by the traits of sometimes distant foveal elements.

Finally, UI may be due to perceptual inflation, whereby apparent detail in the periphery is not sustained on perceptual content, but due to decisional or metacognitive biases (Odegaard, Chang, Lau, & Cheung, 2018). During the control session in our experiment, where a physically uniform pattern was presented at times, participants were less prone to report UI during presentation of the nonuniform pattern compared with the illusion session: This suggests a shift in decision criterion for uniformity. Importantly, these processes are not exclusive: Possibly both texture processing and perceptual inflation contribute to UI. Further studies may elucidate the precise contribution of the different perceptual mechanisms that underlie foveal-peripheral integration, as demonstrated by UI, and that are central to naturalistic visual experience. However, our results clearly demonstrate that, at least for orientation, these mechanisms do not alter neural coding at the primary visual cortex.

## Supplemental Material

Supplemental material for The Illusion of Uniformity Does Not Depend on the Primary Visual Cortex: Evidence From Sensory Adaptation Click here for additional data file. Supplemental material for The Illusion of Uniformity Does Not Depend on the Primary Visual Cortex: Evidence From Sensory Adaptation by Marta Suárez-Pinilla, Anil K. Seth and Warrick Roseboom in i-Perception
