# Supplementary material for: The Illusion of Uniformity Does Not Depend on the Primary Visual Cortex: Evidence From Sensory Adaptation
Source: Iperception. 2018 Sep 27;9(5):2041669518800507. doi: 10.1177/2041669518800728 (PMC6166314; doi:10.1177/2041669518800728)
Supplement: Supplemental material for The Illusion of Uniformity Does Not Depend on the Primary Visual Cortex: Evidence From Sensory Adaptation [file Supplemental_material.pdf]

Supplementary Materials

S1. EXPERIMENTAL DATA

The supplementary file 'data_table.csv' contains the data from all 30 participants - each row corresponds to a single trial. Each participant contributed with 6 experimental blocks during the Illusion session and as many during the Control session; each block had 24 trials. However, we excluded from the analysis all blocks wherein exposure to the adapting pattern was attained for less than 2/3 of the adaptation phase (<120 seconds), due to improper gaze fixation. To ensure that both sessions were a reflection of each other for each participant, homologous blocks to those excluded in the other session were also excluded. Finally, since our analyses involved comparison of response patterns across adapting conditions CX and XC, we excluded 2 participants wherein all valid (i.e. previously not excluded) blocks were of one single condition. In total, 23 participants and 5647 trials entered the analysis.

The detailed variables are as follows:

1. ID: the participant's ID number (1-30).
2. Session: 1-Illusion session, 2-Control session.
3. Block: each session has 6 experimental blocks.
4. Adapting condition: 1-CX (local CW, global XCW), 2-XC (local XCW, global CW) -see main article for details.
5. Trial: numbered 1-24 for each block.
6. Test orientation: the orientation (or tilt) of the test Gabor presented in that trial.
7. Responded (Y/N): indicates whether the trial was responded within the maximum 7.5 seconds allowed. 1-trial responded, 0-trial missed. In our experiment, there were 55 missed trials (0.64%).
8. XCW response (Y/N): 1-XCW response, 0-CW response. Missed trials take a NaN value.
9. Time of adaptation: time (in seconds) of effective exposure to the adapting pattern during that block's adaptation phase. The total duration of the adaptation phase is 180 seconds, but the pattern is removed from the screen when centred gaze fixation is not attained.
10. Proportion of uniformity: proportion of time of uniformity over the total time of effective pattern presentation during that block's adaptation phase; ranges from 0 to 1. In the Illusion session, this variable refers to reported illusory uniformity; during the Control session it applies to physical uniformity.
11. Sufficient adaptation: 1-the current block, as well as its homologous in the other session, have both at least 120 seconds of pattern presentation during the adaptation phase. 0-the block is excluded from the analysis since it does not meet the above conditions.
12. Counterbalanced conditions. Only applies to blocks with value 1 in the 'Sufficient adaptation' variable: 1-the current participant has non-excluded blocks with the two contrasted adapting conditions (CX, XC) in each session. 0-due to block exclusion, the current participant does not have valid data of

the two conditions; therefore, the participant's data is not suitable for analyses that directly compare response pattern across conditions.

S2. PSYCHOMETRIC CURVE FITTING

2.1. Individual Psychometric curves

Figure S1 shows the individual responses (proportion of XCW reports by test Gabor orientation) and best-fitting cumulative Gaussian functions obtained for each participant, with their data split by adapting condition (CX and XC) and session (Illusion and Control). Only the 23 participants with valid data on both adapting conditions are presented. The methodology for cumulative Gaussian fitting is detailed in the Methods section of the main article ('Statistical analysis' subheading).

This figure is also included in full size as a Supplementary file and can be produced with the included Matlab script 'UI_analysis.m'.

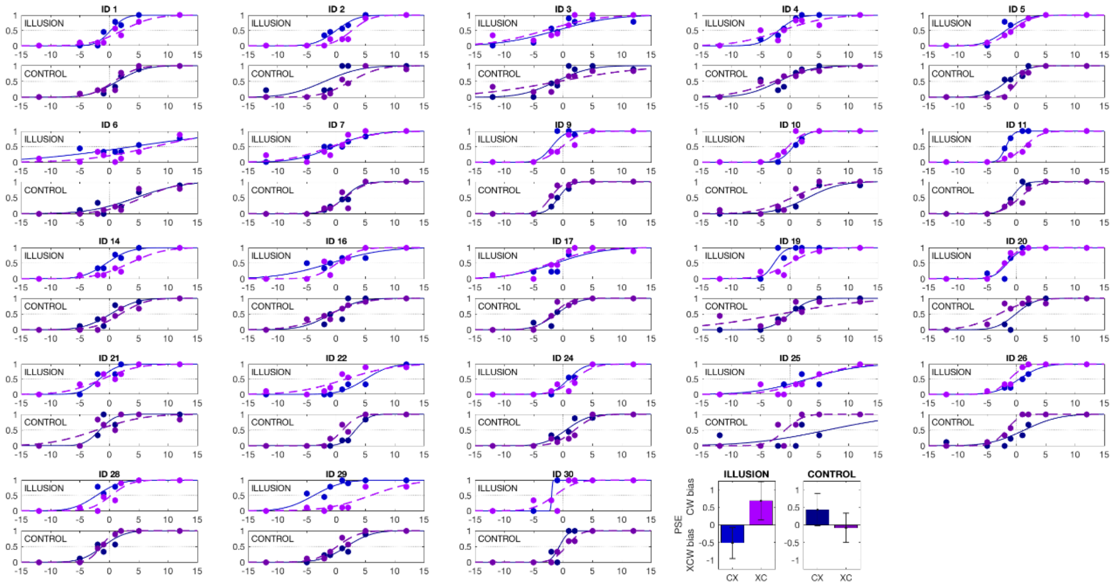

Figure S1. Responses and Psychometric function: individual results. Response pattern and best-fitting Psychometric curve for each participant's data, split by adapting condition and session. As indicated in the figure, the upper plot for each participant corresponds to the Illusion session and the lower one to the Control session. In all plots (except for the summary graph at the bottom right), the X axis represents the test Gabor orientation, ranging from -12° to 12°, with negative and positive sign conventionally indicating CW (clockwise) and XCW (counter-clockwise) tilt, respectively. The Y axis represents the proportion of 'XCW' reports per test Gabor orientation. Blue and purple dots pertain to adapting conditions CX (local orientation CW, global XCW) and XC (local XCW, global CW), respectively. The dotted lines present the best-fitting cumulative Gaussian curve for the participant's data, split by session and adapting condition. A rightward shift of one curve with respect to the other condition (or session) indicates a comparatively more CW bias, and vice versa. In other words, a positive PSE indicates a CW bias and a negative PSE a XCW bias. The summary graph at the bottom right indicates the average of the individual PSEs of all the Psychometric curves shown in the previous plots, separated by session (Illusion session in the left plot and Control session in the right plot) and adapting condition (as labelled in the horizontal axis: CX/XC). The error bars indicate the standard error. For the Illusion session, the negative and positive PSE in conditions CX and XC, respectively, indicate that the tilt after-effect (TAE) is driven by (i.e. away from) the local, physical orientation. For the Control session, we observe, on average, responses show a trend toward a global-driven TAE in condition CX and are unbiased in XC. These results likely show a 'mixture' of local and global-driven TAE. In the Control session, the adapting Gabor takes the global orientation during the times

of presentation of a physically uniform pattern. Thus, results in this session are also compatible with physical-driven adaptation.

2.2. Goodness-of-Fit

We assessed the goodness-of-fit separately for each participant and session, by using the function provided to such effect in the Palamedes toolbox (goodness-of-fit test across several conditions, specifically CX and XC conditions). The target model assumed a cumulative Gaussian curve for the relationship between test Gabor orientation and proportion of XCW reports, with varying thresholds (α) and slopes (β) across adapting conditions, and a guess rate (γ) and lapse rate (λ) fixed to zero in both conditions. Table S1 shows the pDev values, indicative of the goodness-of-fit of the target model to the participant's data. The fit was reasonable (pDev≥ 0.05) for most datasets (37/46), and poor only for 9/46. On average, $pDev_{Illusion}$= 0.282 (SE 0.055) and $pDev_{Control}$= 0.213 (SE 0.031) were well above the 0.05 cut-off point; according to a Bayesian paired-samples t-test, there was no evidence for a difference between the goodness-of-fit of the Illusion compared to the Control session (BF_{10}=0.365), with an anecdotal, but tending toward moderate (BF_{10}<=1/3) support for the null hypothesis (i.e. equal goodness-of-fit across sessions).

ID	$pDev_{Illusion}$	$pDev_{Control}$
1	0.307	0.328
2	0.515	0.000
3	0.035	0.014
4	0.416	0.320
5	0.372	0.401
6	0.242	0.180
7	0.071	0.247
9	0.007	0.397
10	0.099	0.016
11	0.714	0.337
14	0.866	0.300
16	0.331	0.310
17	0.000	0.182
19	0.079	0.115
20	0.037	0.096
21	0.654	0.037
22	0.172	0.302
24	0.086	0.227
25	0.650	0.092
26	0.565	0.006
28	0.075	0.250
29	0.125	0.539
30	0.071	0.194

Table S1. Goodness-of-fit for each participant's and session's data. pDev value ranges between 0 and 1: the larger this value, the better the fit.

Thus, the Psychometric function goodness-of-fit to the data is acceptable on average, with only a few individuals who exhibit a poor fit. In the following sub-section (2.3.) we show an almost perfectly linear correlation between measures directly based on participants' responses and measures based on fitted Psychometric functions (PSE), further confirming that the latter measures are accurately tracking each participant's response patterns. Besides, in Section S4 of the Supplementary Materials, we additionally show that analyses based directly on responses render analogous results to those based on PSE that are reported in the main article.

2.3. Correlation between individual response-based measures (%XCW) and Psychometric function-based measures (PSE)

In this section we aimed to confirm that the PSEs obtained from the participants' Psychometric functions, which are the key measures to the analyses reported in the main article, were accurately tracking the participants' response bias per condition and session.

Consequently, we defined the variable %XCW, referring to the proportion of 'counter-clockwise' (XCW) reports per participant, session and adapting condition. A participant may report either 'CW' (clockwise) or 'XCW' (counter-clockwise) for every presented test Gabor. Note that test Gabor clockwise and counter-clockwise orientations are equally frequent per block, with an average orientation of 0° for all sessions and adapting conditions. Therefore, in absence of any response bias we should expect %XCW=50%, corresponding to PSE=0°. A lower proportion (%XCW<50%) would indicate a CW bias; which would be reflected by a PSE>0 (given the conventional negative and positive sign for CW and XCW orientations, respectively). Conversely, both %XCW>50% and PSE<0 indicate XCW bias.

Therefore, if the PSEs derived from Psychometric curve fitting are an accurate measure of each participant's response bias (per condition and session), we should observe a negative correlation between individual %XCW and PSE. Table S2 shows that this is the case, demonstrating almost perfect negative correlations for all conditions and sessions (r<-0.95). The individual results utilised for computed the correlations are detailed in Supplementary section S3.

Measurements	Pearson's r	95% Credible Interval		BF_{-0}
$\%XCW_{Illusion\ CX}$, $PSE_{Illusion\ CX}$	-0.962	-0.983	-0.896	$1.135*10^{11}$
$\%XCW_{Illusion\ XC}$, $PSE_{Illusion\ XC}$	-0.961	-0.982	-0.892	$7.929*10^{10}$
$\%XCW_{Control\ CX}$, $PSE_{Control\ CX}$	-0.955	-0.980	-0.879	$2.372*10^{10}$
$\%XCW_{Control\ XC}$, $PSE_{Control\ XC}$	-0.973	-0.988	-0.925	$3.349*10^{12}$

Table S2. Bivariate correlations between individual %XCW and PSE, computed separately for each session (Illusion/Control) and adapting condition (CX/XC). An almost perfect negative correlation is observed in all cases, indicating that PSE is accurately tracking the participant's response bias. BF_{-0} refers to the Bayes factor for the existence of a negative correlation (extreme evidence in its favour in all cases).

Like PSE, %XCW indicates whether responses are biased to the CW or XCW orientation. However, for summarizing responses across different conditions (CX, XC), we needed to define more abstract measures that expressed whether the TAE was driven by (i.e. away from) the local or the global orientation of the adapting pattern.

In the main article we defined $dPSE=PSE_{CX}-PSE_{XC}$: dPSE<0 would indicate a local-driven TAE and dPSE>0 a global-driven TAE. Here, we further defined %Local as the proportion of responses in the same direction as the 'local' orientation of the block: i.e., the proportion of CW reports in CX adapting condition (100-%XCW), and the proportion of XCW reports (%XCW) in XC condition. A %Local<50% would indicate a local-driven TAE (responses are biased away from the local orientation), and %Local>50% would reflect a global-driven TAE.

Therefore, in this case we should expect a positive correlation between response-based measures (%Local) and Psychometric curve-based measures (dPSE). This result is shown in Table S3 and Figure S2, again demonstrating the validity of dPSE as a measure of response bias.

**Measurements**	**Pearson's r**	**95% Credible Interval**		**BF_{+0}**
$\%Local_{Illusion}$, $dPSE_{Illusion}$	0.955	0.875	0.981	$7.334*10^9$
$\%Local_{Control}$, $dPSE_{Control}$	0.946	0.851	0.976	$1.208*10^9$

Table S3. Correlations between individual %Local and dPSE, computed separately for each session (Illusion/Control). An almost perfect positive correlation is observed, indicating that dPSE is accurately tracking the participant's response bias. BF_{+0} refers to the Bayes factor for the existence of a positive correlation (extreme evidence in both cases).

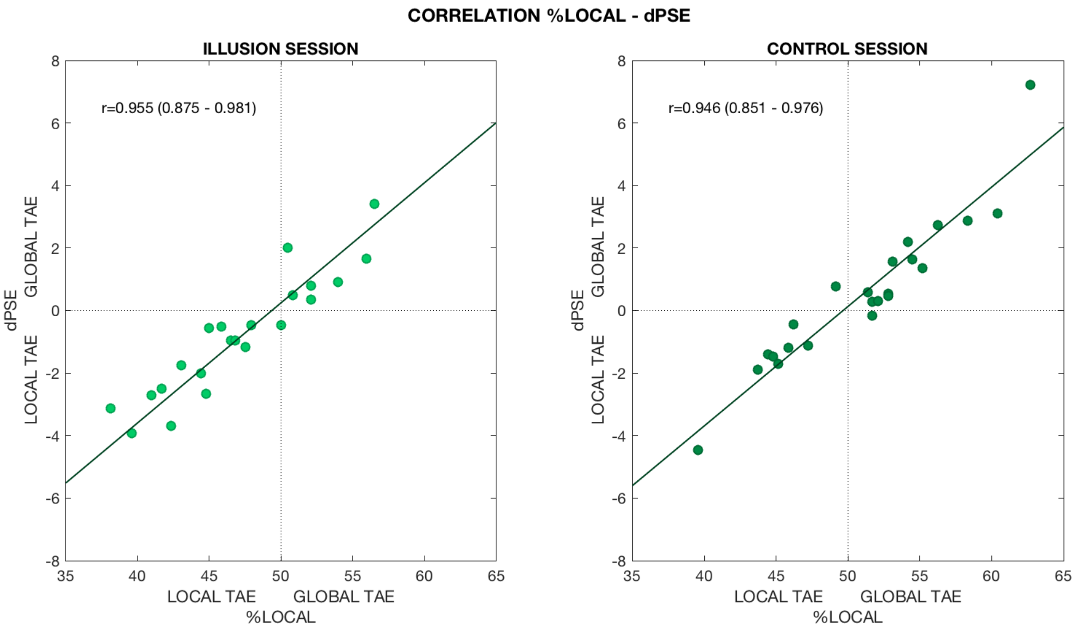

Figure S2. Correlations between individual %Local and dPSE, computed separately for each session (Illusion/Control). An almost perfectly linear positive correlation is observed, indicating that dPSE is accurately tracking the participant's response bias.

S3. INDIVIDUAL SUMMARY MEASURES

3.1. Individual results: proportion of XCW reports (%XCW) and PSE per adapting condition and session

Table S4 presents the average proportion of XCW reports (%XCW) for each participant (N=25), session (Illusion/Control) and adapting condition (CX/XC), alongside the corresponding PSEs derived from Psychometric curve fitting (see Supplementary section S2).

Both %XCW and PSE can be used to ascertain the existence of a CW (%XCW<50%, PSE>0) or XCW bias (%XCW>50%, PSE<0). The neutral value, indicating overall absence of systematic response bias, corresponds to %XCW=50% and PSE=0º.

This table contains all the data from blocks with sufficient (≥120 s) time of pattern exposure during the adaptation phase: i.e., all blocks where the pattern-driven tilt after-effect (TAE) could be adequately probed. As stated above, two participants (ID12 and ID18) only had valid data from one of the adapting conditions and were thus excluded from summary analyses. Nevertheless, in the Supplementary section S5, we show that summary statistics for all 25 participants with valid blocks are very similar to the 23 participants with counterbalanced conditions.

ID	%XCW CX Illusion	%XCW XC Illusion	%XCW CX Control	%XCW XC Control	PSE CX Illusion	PSE XC Illusion	PSE CX Control	PSE XC Control
1	48.611	37.500	41.667	44.444	0.222	2.220	1.334	0.754
2	50.000	31.944	58.333	37.500	0.000	2.700	-2.008	2.443
3	55.556	59.524	57.729	52.083	-1.117	-3.123	-1.225	-0.780
4	62.500	56.250	59.722	62.500	-1.921	-1.362	-1.910	-2.691
5	62.500	55.556	61.111	48.611	-1.832	-0.867	-1.677	0.211
6	40.278	25.000	30.556	25.000	3.835	7.529	4.501	5.627
7	52.083	52.778	40.278	39.583	-0.399	-0.892	1.324	1.499
9	65.278	51.389	56.280	66.667	-1.932	-0.175	-0.771	-2.133
10	44.867	52.778	34.722	47.222	0.578	-0.332	3.061	0.322
11	64.734	41.002	56.944	45.833	-1.821	1.297	-0.846	0.545
12	47.917	N/A	43.750	N/A	0.374	N/A	1.352	N/A
14	52.778	31.944	48.611	38.889	-0.468	3.451	0.218	1.921
16	54.167	48.611	54.167	55.556	-0.979	0.177	-0.828	-1.107
17	55.556	59.722	56.944	62.500	-1.269	-2.062	-1.134	-1.681
18	N/A	58.333	N/A	50.000	N/A	-1.239	N/A	-0.155
19	70.833	50.000	50.000	54.167	-2.710	-0.057	0.000	-1.564

20	60.417	64.583	50.000	66.667	-1.534	-1.877	-0.128	-3.010
21	62.500	56.250	62.500	60.417	-1.718	-1.242	-1.624	-2.098
22	23.279	43.056	27.083	41.667	4.982	1.567	3.309	1.110
24	43.056	36.715	47.162	36.775	0.926	1.881	0.511	1.980
25	37.500	33.333	29.167	54.545	2.939	3.411	6.434	-0.795
26	46.377	58.333	41.667	62.500	0.436	-1.228	1.356	-1.761
28	63.889	47.222	56.944	61.111	-2.182	0.311	-1.098	-1.408
29	70.833	25.000	39.614	48.611	-3.822	5.913	1.786	0.156
30	66.667	58.333	54.167	45.833	-1.942	-1.435	-0.625	0.559

Table S4. Proportion (%) of XCW report and PSE per participant (N=25), session (Illusion/Control) and adapting condition (CX/XC).

3.2. Individual results: relationship between time of uniformity and direction of the TAE

Table S5 presents summary measures for the overall direction of the TAE per participant and session, alongside the time of perceived (Illusion session) and physical (Control session) uniformity.

As described in Supplementary sections 2.3 and 4.1, %Local indicates the proportion of reports in the same direction as the block's local orientation: %Local<50% would indicate a TAE away from the local orientation, and vice versa. In the main article we defined dPSE=PSE_{CX}-PSE_{XC}; dPSE<0 indicates a local-driven TAE, and vice versa. The time of uniformity is expressed in terms of the proportion (%) of uniformity for the total time of effective exposure to the adapting pattern during the adaptation phase.

The table presents all 25 participants with valid experimental blocks.

ID	%Local Illusion	dPSE Illusion	%Time Perceived Uniformity	%Local Control	dPSE Control	%Time Physical Uniformity
1	44.444	-1.999	20.876	51.389	0.580	21.779
2	40.972	-2.700	2.456	39.583	-4.451	2.432
3	50.476	2.007	71.867	46.196	-0.445	71.149
4	45.000	-0.559	22.830	49.167	0.782	22.529
5	46.528	-0.964	1.360	43.750	-1.888	1.375
6	42.361	-3.694	20.347	47.222	-1.126	20.156
7	50.833	0.493	0.557	51.667	-0.175	0.575
9	43.056	-1.756	10.424	55.193	1.362	10.720
10	53.955	0.910	37.543	56.250	2.739	36.389
11	38.134	-3.118	17.310	44.444	-1.390	17.418
12	52.083	N/A	51.639	56.250	N/A	50.622
14	39.583	-3.919	11.351	45.139	-1.703	11.192
16	47.500	-1.156	36.634	51.667	0.279	36.466

17	52.083	0.793	37.508	52.778	0.547	37.267
18	58.333	N/A	5.846	50.000	N/A	6.339
19	44.792	-2.653	50.189	53.125	1.564	51.533
20	52.083	0.343	50.943	58.333	2.882	51.291
21	50.000	-0.477	15.207	52.778	0.473	16.411
22	56.522	3.415	6.177	54.167	2.199	5.626
24	46.830	-0.954	15.723	44.807	-1.469	15.770
25	47.917	-0.472	58.514	62.689	7.229	58.557
26	55.978	1.664	5.739	60.417	3.117	5.672
28	41.667	-2.493	53.061	52.083	0.310	52.843
29	27.083	-9.735	65.333	54.499	1.630	65.671
30	45.833	-0.507	3.541	45.833	-1.184	3.649

Table S5. %Local, dPSE and average time (%) of uniformity per participant and session (Illusion/Control). Time of perceived (Illusion session) and physical (Control session) uniformity are defined as the % (calculated per block) of time of uniformity for the entire time of presentation of the adapting pattern: thus, it may range between 0 and 100.

S4. ANALYSES BASED ON RESPONSE-BASED MEASURES

In this section we reanalyse the dataset in an analogous manner to the main text, but basing our analyses in the actual participants' responses rather than in the fitted Psychometric functions. We demonstrate a neat convergence between both approaches at every step of the analysis.

4.1. Response-based measures: %XCW and %Local

As described in the main article, our analyses aimed to ascertain whether or not the illusory orientation seen under UI was able to cause a TAE. Thus, we analysed the PSE for each adapting condition (PSE_{CX} and PSE_{XC}) and defined a summary measure, $dPSE=PSE_{CX}-PSE_{XC}$, whose sign would indicate the overall direction of the TAE: dPSE<0 indicated a local-driven TAE (corresponding to physical-driven adaptation in the Illusion session) and dPSE>0 a global-driven TAE (corresponding to adaptation to the illusion during the Illusion session, and to the physical replication of the Illusion during the Control session).

Analogous measures to PSE_{CX}/PSE_{XC} and the more abstract dPSE can be constructed on the basis of the actual responses, rather than the fitted curves. Likewise, predictions for both competing hypotheses (UI is not/is able to produce TAE) can be made regarding analyses on these measures. See Supplementary sections 2.3, 3.1 and 3.2.

1. $\%XCW_{CX}$ and $\%XCW_{XC}$: the proportion (%) of 'counter-clockwise' reports in adapting conditions CX and XC, respectively. If the TAE biases responses away from the local orientation, we would expect $\%XCW_{CX}>50\%$ and $\%XCW_{XC}<50\%$; reverse predictions are made for global-based TAE. Therefore, as described in the Supplementary section 2.3., there is a negative

correlation between %XCW and PSE, both conceptually and in the actual data.

2. %Local: proportion (%) of reports in the same direction as each block's local orientation (100-%XCW for CX blocks, %XCW for XC blocks). As dPSE, %Local indicates the overall direction of the TAE: %Local<50% indicates a local-driven TAE, while %Local>50% indicates a global-driven TAE. In this case, the correlation between both measures (conceptually and in the actual data) is positive.

4.2. <u>Overall results per condition and session</u>

Figure S3 presents the average proportion of XCW responses per adapting condition and session, along with the corresponding average PSEs. The latter are also presented in the Figure 2 of the main manuscript.

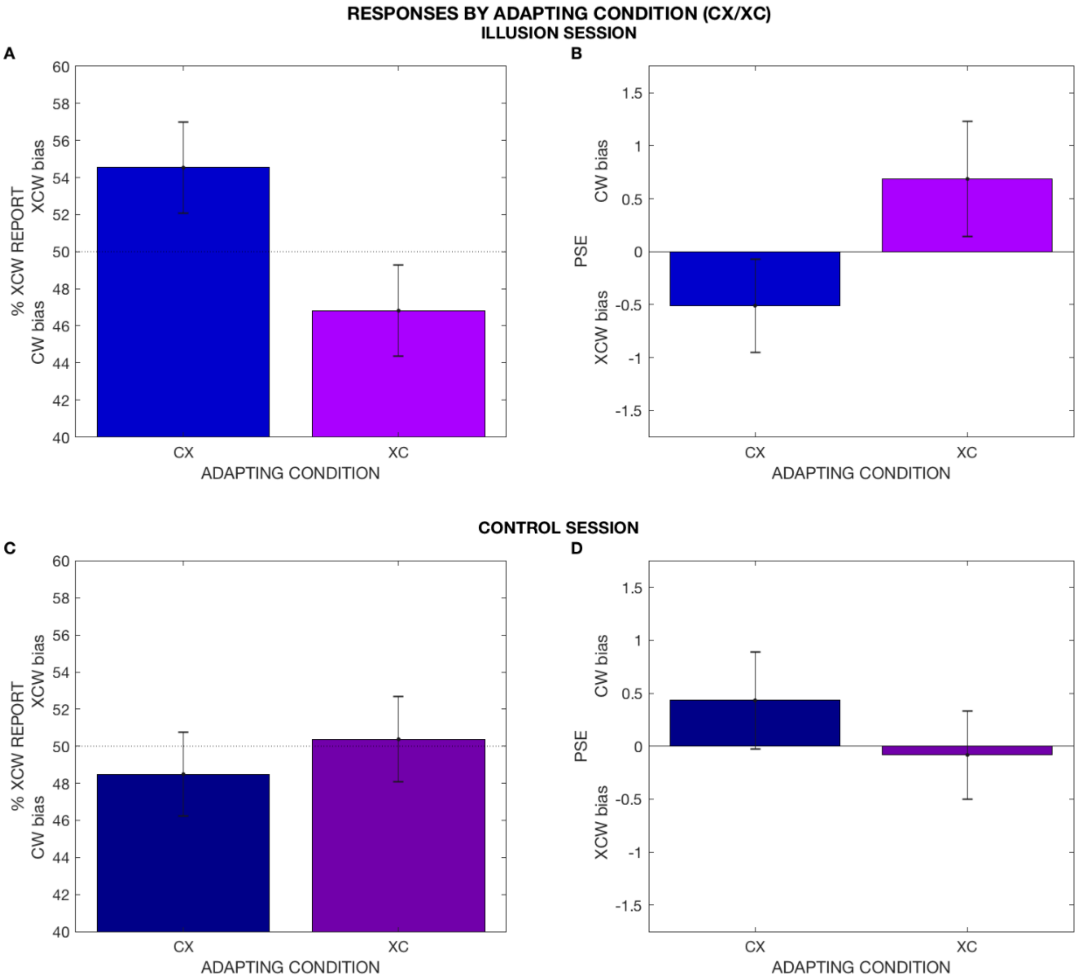

Figure S3. Response patterns by adapting condition: Illusion (3A-3B) and Control (3C-3D) session. **3A** and **3C** present the average %XCW computed separately by participant, adapting condition and session. The error bars represent the between-participant standard error. **3B** and **3D** depict the average point of subjective equality (PSE) computed separately per participant and condition. **3A-B.** Illusion session. The %XCW and PSEs for both adapting conditions reflect a bias away from local orientation (local-driven TAE). **3C-D.** Control session. On average, responses show a trend toward a global-driven TAE in condition CX and are unbiased in XC. These results show that perceived (illusion) and physical (control) uniformity behave differently, suggesting that the TAE is always driven by the physical orientation, even when that orientation is unseen under UI.

4.2.1. Illusion session

During the Illusion session, on average, $\%XCW_{CX}$=54.533% and $\%XCW_{XC}$=46.818% reflected XCW and CW bias, respectively ($\%XCW_{CX}>\%XCW_{XC}$ Bayesian paired-samples t-test: BF_{0+}=7.244). In other words, reports were biased *away* from the local orientation in both conditions (%Local =46.245%).

These results are convergent with PSE-based results presented in the main manuscript: PSE_{CX}=-0.502°, PSE_{XC}=0.687°, dPSE=-1.197°, $PSE_{CX}<PSE_{XC}$ Bayesian paired-samples t-test: BF_{-0}=3.057. Both approaches reflect a local, physical-driven adaptation, with no trace of UI-induced TAE.

4.2.2. Control session

Results for the Control session indicated a global-driven bias in condition CX and absence of noticeable bias in XC (the latter likely representing a mixture of local and global-driven bias, since physical uniformity is presented during only part of the time). The proportion of reports in the direction of each block's local orientation ($\%Local_{CO}$) was 51.007%, compared with 46.245% during the Illusion session. Bayesian t-test showed strong evidence for $\%Local_{IL}<\%Local_{CO}$: BF_{-0}=12.862. Results comparing dPSE across sessions were in the same direction: $dPSE_{CO}$=0.516°, compared with $dPSE_{IL}$=-1.197°, Bayesian paired t-test for $dPSE_{IL}<dPSE_{CO}$ rendered BF_{-0}=7.476.

Thus, results showed that illusory and physical uniformity presented for the same time had different effects, with the introduction of physical uniformity at times during the Control session producing a shift toward more global-based TAE.

4.3. <u>Time-dependent results per session</u>

Supplementary figure S4 presents the individual %Local as a function of the time of perceived/physical uniformity during the Illusion and Control session, respectively, alongside the time-dPSE correlations, also for each session, that have been presented in the main article.

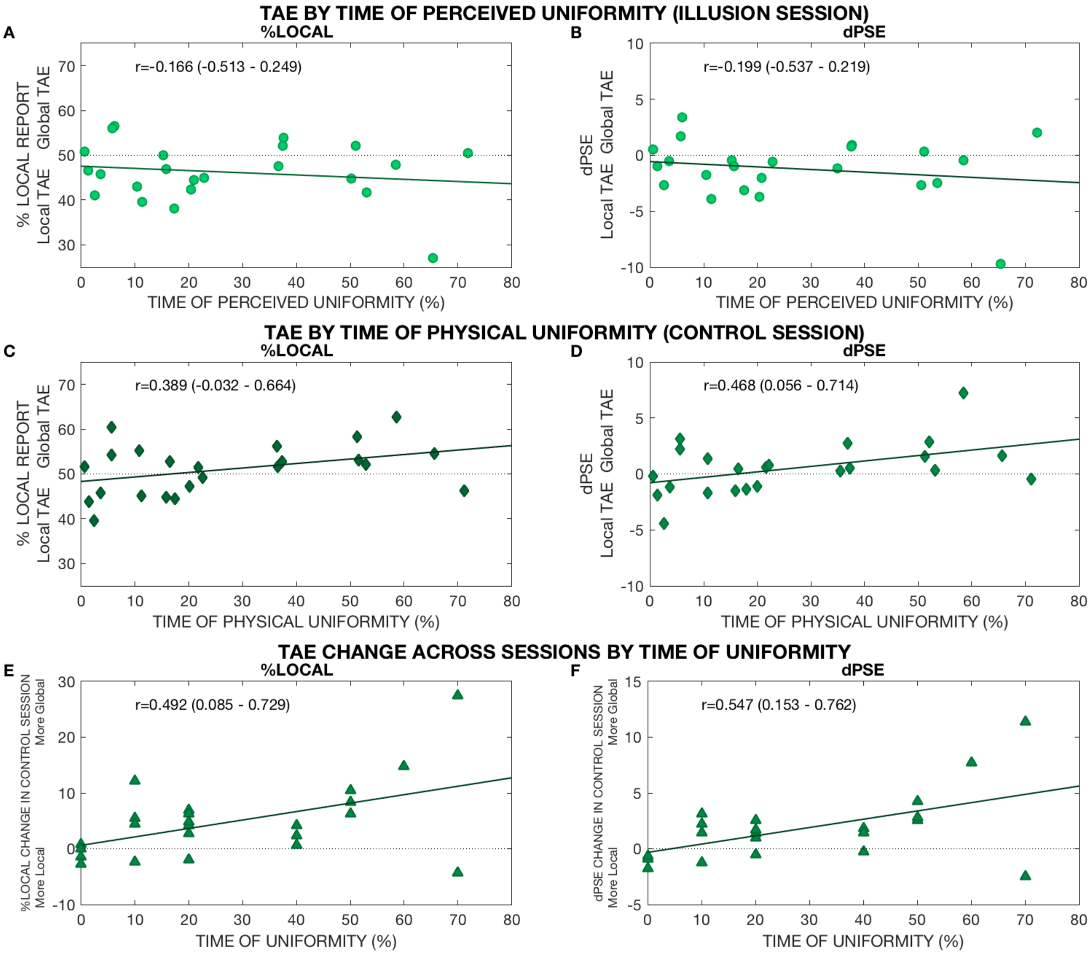

Figure S4. TAE by time of uniformity. 4A-4B: Correlation between time (%) of perceived uniformity and overall direction of the TAE (local/global driven) during the Illusion session, expressed in terms of %Local (4A) and dPSE (4B). Each data point represents a participant's average data during the session. Pearson's correlation coefficient and 95% credible intervals are in each panel, showing that there is no significant time-dependency for perceived uniformity. **4C-4D**: Correlation between time (%) of physical uniformity and overall direction of the TAE (local/global driven) during the Control session, expressed in terms of %Local (4C) and dPSE (4D). Each data point represents a participant's average data during the session. Pearson's correlation coefficient and 95% credible intervals are in each panel, showing a positive time-dependency of physical uniformity in the expected direction: presentation of the global panel for a longer time leads to more global-driven TAE, unlike the lack of effect of illusory uniformity during the Illusion session. **4E-4F.** TAE change by time of uniformity. The horizontal axis represents each participant's time (%) of Uniformity. For illustrative purposes, it has been rounded to the nearest 10%, so that the horizontal axis is applicable to both sessions of each participant (since physical uniformity is presented for the same time than the duration of the illusion reported in the previous session, but in practice the gaze-contingent stimulus removal during the Control session generates a slight jitter). The vertical axis represents the change in the summary measure of the direction of the TAE (%Local in 4E, dPSE in 4F) in the Control session with respect to the Illusion session: i.e., in 4E, the vertical axis represents $\%Local_{Control}$ - $\%Local_{Illusion}$ and in 4F it represents $dPSE_{Control}$ - $dPSE_{Illusion}$. A positive difference indicates that a participant's responses during the Control session are subject to a global-driven bias (higher %Local/dPSE) compared to the Illusion session. Both sessions are identical for each participant, except that uniformity is illusory during the Illusion session, and physical during the Control session. We observe that, for short times of uniformity, a participant's response pattern is almost identical in both sessions (roughly 'zero' difference). However, as time of uniformity increases, the patterns become more different, with the Control session showing a more global-driven bias. Pearson's correlation coefficient confirms this positive correlation, with a BF_{+0}=7.458 for time - %Local change and a BF_{+0}=15.997 for time – dPSE change.

4.3.1. Illusion session

We ran a Bayesian bivariate correlation between each participant's average time of perceived uniformity and %Local during the Illusion session, showing a Pearson's

r=-0.166 (95% credible intervals -0.513 – 0.249), with moderate evidence against a positive correlation: BF_{+0}=0.158. See figure S4A. This result was convergent with the lack of correlation between time and dPSE (for the Illusion session), as reported in the main article and presented in figure S4B. Thus, the direction of the TAE is independent of the time of perceived uniformity, in agreement with the hypothesis that illusory orientation is not able to produce TAE.

4.3.2. Control session

A bivariate correlation between time (%) of physical uniformity and %Local rendered a Pearson's r=0.389 (95% credible intervals -0.032 – 0.664), suggesting a positive correlation, although with only anecdotal evidence: BF_{+0}=2.421 -see Figure S4C. These results are in agreement with time-dPSE correlation during the Control session (although for the effect reported in the main text there was moderate evidence supporting a positive correlation: BF_{+0}=5.546) – see Figure S4D. In other words, evidence supported a time-dependency for the presentation of physical uniformity and the appearance of a global TAE, ruling out that the lack of correlation in the Illusion session was due to all participants experiencing UI for an insufficient time to exhibit adaptation effects.

Further evidence about the effect of physical, compared to perceived uniformity, is shown in Figures S4E and S4F. These panels show the correlation between each participant's time of uniformity (rounded to the nearest 10% so that the position along the horizontal axis applies to both the Illusion and Control session, since the gaze-contingent mechanism introduces some variation) and the change in the direction of the TAE in the Control session, compared to the same participant's responses during the Illusion session: $\%Local_{Control}$ - $\%Local_{Illusion}$ in S4E, $dPSE_{Control}$ - $dPSE_{Illusion}$ in S4F. A positive difference indicates that a participant's responses during the Control session are subject to a global-driven bias (higher %Local/dPSE) compared to the Illusion session. A negative difference indicates the opposite. The trial sequence in the blocks of both sessions is identical: only the replacement of illusory by physical uniformity during the adaptation phase distinguishes the Control from the Illusion session. We observe that, for short times of uniformity, a participant's response pattern is almost identical in both sessions (roughly 'zero' difference). However, as time of uniformity increases, the patterns become more different, with the Control session showing a more global-driven bias. Pearson's correlation coefficient confirms this positive correlation: for time and %Local change, r=0.492 (95% credible intervals: 0.085 – 0.729), with a BF_{+0}=7.458; for time and dPSE change, r=0.547 (0.153 – 0.762), BF_{+0}=15.997.

S5. COMPARISON OF RESULTS FOR THE ENTIRE SAMPLE (N=25) AND FOR THE SAMPLE WITH COUNTERBALANCED ADAPTING CONDITIONS (N=23)

Because our analyses involved comparison of response patterns across adapting conditions (CX/XC), we excluded data from two participants who had valid blocks of a single condition. Here we show that the summary results between the entire

sample (N=25) and the counterbalanced sample (N=23) are comparable. Table S6 summarizes %XCW and table S7 the PSE per condition and session.

**Sample**	**%XCW$_{CX\ Illusion}$**	**%XCW$_{XC\ Illusion}$**	**%XCW$_{CX\ Control}$**	**%XCW$_{XC\ Control}$**
N=25	54.257 (SE 2.266)	47.298 (SE 2.313)	48.297 (SE 2.087)	50.362 (SE 2.106)
N=23	54.533 (SE 2.397)	46.818 (SE 2.414)	48.495 (SE 2.214)	50.377 (SE 2.243)

Table S6. Descriptive statistics (sample mean and standard error SE) for the proportion of XCW reports per adapting condition and session, when considering the entire sample (N=25) or only those 23 participants with valid blocks from both adapting conditions. Note than, since a participant (ID18) lacks blocks of condition CX and another (ID12) lacks data of condition XC, the number of data points contributing to the descriptive results for the entire sample (N=25) is only 24 for each case, but the extra participant is different for each condition.

**Sample**	**PSE** **CX Illusion**	**PSE** **XC Illusion**	**PSE** **CX Control**	**PSE** **XC Control**
N=25	-0.473 (SE 0.405)	0.607 (SE 0.507)	0.471 (SE 0.422)	-0.086 (SE 0.383)
N=23	-0.502 (SE 0.430)	0.687 (SE 0.534)	0.433 (SE 0.448)	-0.083 (SE 0.408)

Table S7. Descriptive statistics (sample mean and standard error SE) for the PSE per adapting condition and session, when considering the entire sample (N=25) or only those 23 participants with valid blocks from both adapting conditions. Note than, since a participant (ID18) lacks blocks of condition CX and another (ID12) lacks data of condition XC, the number of data points contributing to the descriptive results for the entire sample (N=25) is only 24 for each case, but the extra participant is different for each condition.
